# Modeling growth of *Lactobacillus helveticus* and *Kluyveromyces marxianus* isolated from fermented camel milk (shubat)

**DOI:** 10.3389/fmicb.2026.1913745

**Published:** 2026-09-03

**Authors:** Aikerim Ospanova, Askhat Khalbayev, Shynar Akhmetsadykova, Nazerke Begdildayeva, Zauresh Bilal, Arailym Akhatzhanova, Marko Vinceković, Askar Kondybayev

**Affiliations:** 1 Department of Biotechnology, M. Auezov South Kazakhstan University, Shymkent, Kazakhstan; 2 Department of Biotechnology, LLP “Research and Production Enterprise” “Antigen, ” Almaty, Kazakhstan; 3 Department of Biotechnology, Farabi University, Almaty, Kazakhstan; 4 Division of Agroecology, Department of Chemistry, Faculty of Agriculture, University of Zagreb, Zagreb, Croatia; 5 Department of Chemical and Biochemical Engineering, Satbayev University, Almaty, Kazakhstan

**Keywords:** fermentation kinetics, fermented camel milk, *Kluyveromyces marxianus*, LAB–yeast coculture, *Lactobacillus helveticus*, microbial growth modeling, shubat

## Abstract

Shubat is a traditional fermented camel milk beverage in which lactic acid bacteria and yeasts jointly determine fermentation behavior, but the kinetic properties of autochthonous strains remain insufficiently characterized. This study aimed to model the growth and metabolite production of *Lactobacillus helveticus* SH4 and *Kluyveromyces marxianus* Y45, two most dominant species found in Kazakhstani shubat. Growth was evaluated under different temperatures, pH values, and carbohydrate sources, and described using logistic, cardinal pH—temperature, and Monod models. Lactic acid and ethanol production were measured during cultivation, and co-culture behavior was assessed in modified MRS medium and reconstituted camel milk. *L. helveticus* SH4 showed optimal growth at pH 7.22 ± 0.11 and 39.66 ± 0.38°C, while *K. marxianus* Y45 grew optimally at pH 5.39 ± 0.23 and 37.78 ± 0.71°C. SH4 grew efficiently on glucose, galactose, and lactose, whereas Y45 showed the highest growth rate on glucose. Metabolite analysis indicated lactic acid production by SH4 and ethanol production by Y45. In co-culture, both strains followed monoculture-based growth trends in modified MRS, while reconstituted camel milk reduced the final SH4 population. These results provide quantitative parameters for understanding and improving controlled shubat fermentation.

## 1 Introduction

Fermented foods are created by the action of microbes, which add to the sensory, nutritional, and functional qualities of the finished product. Fermentation in many fermented beverages is driven by the combined activity of multiple types of microorganisms, particularly lactic acid bacteria and yeasts, rather than by a single microbial strain (Belda et al., 2025). These microbes in fermented dairy products can influence acidity, substrate consumption, metabolite production, and product quality.

Shubat, a traditional fermented camel-milk beverage of Central Asia, exemplifies this complexity. Widely consumed across Kazakhstan and adjacent regions of the Turkic dairy belt, it is increasingly recognized as a functional product whose nutritional and immunomodulatory profile derives from a combination of camel-milk-intrinsic bioactives—lysozyme, lactoferrin, immunoglobulins, and insulin-like peptides—and microbially generated metabolites accumulated during fermentation (Bilal et al., 2024). Recent metagenomic and functional-metabolism analyses have refined this picture, demonstrating that shubat harbors a taxonomically rich and functionally specialized consortium in which LAB and fermentative yeasts dominate the active fraction and jointly govern the beverage’s metabolic trajectory (Zhadyra et al., 2025). Shubat is therefore not produced by a single microorganism but through mixed lactic acid and alcoholic fermentation driven by the joint activity of LAB and yeasts within a naturally established microbial coculture (Kochetkova et al., 2022; Seifu, 2023). Among these microorganisms, *Lactobacillus helveticus* was reported as the most abundant bacterial species, whereas *Kluyveromyces marxianus* were identified as the dominant yeast species in Kazakhstani shubat (Bilal et al., 2024).

The biochemical singularity of the camel-milk matrix; however, imposes constraints that distinguish shubat fermentation from its bovine analogs in ways that are only beginning to be quantified. Camel milk exhibits a markedly different casein micelle architecture, a lower κ-casein-total-casein ratio, and elevated concentrations of antimicrobial proteins, all of which translate into distinctive coagulation behavior, atypical acidification curves, and a proteolytic rate up to 1.5-fold higher than that observed in bovine milk under comparable conditions (Ali et al., 2026). These matrix-specific properties have direct kinetic consequences: starter cultures developed and validated for bovine systems frequently exhibit attenuated growth, altered acidification slopes, and inconsistent metabolite yields when transposed to camel milk. Despite this, much of household and small-scale industrial shubat production continues to rely either on spontaneous “back-slopping” of previous batches or on commercial bovine-adapted starters, generating considerable lot-to-lot variability and raising legitimate concerns regarding sensory consistency, textural integrity, and microbial safety (Konuspayeva and Faye, 2021; Alsulami et al., 2025).

Predictive microbiology offers a quantitative technique to describe microbial development during fermentation. Previous research on fermented dairy systems demonstrated that mathematical modeling may be utilized to examine the growth behavior and technical performance of probiotic lactic acid bacteria under regulated settings (Costa et al., 2025). Comparable approaches have been used to describe the survival and physiological state of probiotic strains under processing and gastrointestinal stress, providing a validated template for translating laboratory kinetics into industrially actionable design parameters (Ruarte et al., 2025). For traditional Central Asian fermented dairy products specifically, Kondybayev et al. (2022) demonstrated that classical Gompertz and Baranyi-type formulations could be successfully fitted to *Lacticaseibacillus casei* growth in mare’s milk (koumiss), establishing both methodological precedent and the explicit need for matrix and strain-specific recalibration. The same conceptual scaffolding now underpins emerging work on machine-learning-driven process control and digital-twin architectures for fermented-food manufacturing (Wang et al., 2025).

This study aimed to characterize the growth and metabolic activity of two predominant autochthonous microorganisms isolated from Kazakhstani shubat using an integrated mathematical modeling approach. Although the growth of Lactobacillus helveticus and Kluyveromyces marxianus has been investigated previously, the strain-specific kinetics of shubat isolates and their combined behavior in a camel-milk matrix remain insufficiently characterized. The novelty of this study lies in the kinetic characterization of the shubat-autochthonous strains *L. helveticus* SH4 and *K. marxianus* Y45 and in the use of reconstituted camel milk as the fermentation matrix. In addition, their experimental co-culture behavior was evaluated in modified MRS medium and reconstituted camel milk, a model matrix relevant to shubat fermentation, and compared with monoculture-derived predictions.

## 2 Materials and methods

### 2.1 Microorganisms and identification

*Lactobacillus helveticus* SH4 and *Kluyveromyces marxianus* Y45 were isolated from fermented camel milk, shubat, collected in the Almaty region of Kazakhstan. The bacterial isolate SH4 was identified by 16S rRNA gene sequencing, a commonly used approach for bacterial taxonomic identification (Weisburg et al., 1991), whereas the yeast isolate Y45 was identified by sequencing the internal transcribed spacer region, which is recognized as a standard DNA barcode for fungi (Schoch et al., 2012). The obtained sequences were deposited in the NCBI GenBank database under accession numbers PZ466835 for *L. helveticus* SH4 and PZ466841 for *K. marxianus* Y45. The strains were stored at -80°C in MRS broth medium and Sabouraud broth medium, respectively, supplemented with 30% glycerol.

### 2.2 Culture media composition

Standard de Man, Rogosa, and Sharpe (MRS) broth medium (Himedia, Mumbai, India) was used to cultivate bacteria, whereas Sabouraud broth medium (Himedia, Mumbai, India) was used to cultivate yeast. In substrate-dependent growth tests, glucose was substituted by glucose, galactose, or lactose at the necessary amounts to change the carbon source while maintaining the basal medium composition. This experimental strategy was modified from previously reported methods for the kinetic characterization of lactic acid bacteria and yeasts linked to qymyz (Kondybayev et al., 2023).

Bacto peptone (ThermoFisher, Waltham, MA, United States), glucose, galactose, yeast extract, magnesium sulfate, potassium phosphate dibasic, and ammonium citrate dibasic (Sigma-Aldrich, St. Louis, MO, United States), lactose, sodium acetate anhydrous, manganese sulfate monohydrate, and sodium dihydrogen phosphate (Panreac, Barcelona, Spain) were the reagents used for preparing modified media. In order to assess substrate-dependent growth behavior, carbon sources were selected based on the primary carbohydrates found in fermented milk systems (Ohlsson et al., 2017; Iskandar et al., 2019).

### 2.3 Growth experiments

#### 2.3.1 Temperature’s effect

In Minifor 2BIO fermenters (LAMBDA CZ, s.r.o., Brno, Czech Republic) with an 800 mL working volume, the effects of temperature on the development of the yeast strain and the bacterial strain were assessed separately. An infrared radiator was used to regulate the temperature. Temperatures between 25 and 48°C degrees were used to develop the bacterial strain: 25, 30, 35, 40, 45, and 48 degrees. Temperatures ranging from 20 to 50°C degrees were used to cultivate the yeast strain Y45, namely 20, 25, 30, 35, 38, 40, 45, 48, and 50 degrees. Bacterial and yeast precultures in the stationary phase were prepared by overnight cultivation at 37°C in MRS broth and at 30°C in Sabouraud broth, respectively. The initial population of each strain was adjusted to around 10^6^ CFU⋅mL^−1^. The pH was maintained at 6.6 during bacterial cultivation and 5.6 during yeast cultivation. The pH was automatically regulated using sterile 2 M sodium hydroxide, and agitation was set to 240 rpm. Microbial growth was monitored using an in-line optical turbidity density sensor, Dencytee Arc (Hamilton Bonaduz AG, Bonaduz, Switzerland), and fermentations were carried out until they reached the stationary phase.

#### 2.3.2 Impact of pH and carbohydrate source

The investigations were carried out using the BioTek LogPhase 600 Microbiology Reader (Agilent Technologies, Santa Clara, CA, United States), an automated system for analyzing high-throughput microbial growth curves in aqueous mediums.

With pH values ranging from 3.0 to 12.0 (3.0, 3.5, 4.0, 5.0, 6.0, 7.0, 8.0, 9.0, 10.0, 11.0, and 12.0), maximum specific growth rates were found for both strain at 30°C under similar pH conditions. A range of amounts of glucose, galactose, and lactose (0.5, 1, 5, 10, 20, and 30 g⋅L^−1^) were used to evaluate the impact of restricting carbon sources. The method out-lined by Augustin et al. (1999) was used to calculate maximal specific growth rates from the exponential growth phase, and growth curves were automatically recorded.

#### 2.3.3 Experiments on *L. helveticus* and *K. marxianus* co-culture

Co-culture experiments were conducted using reconstituted camel milk and modified MRS medium. Before inoculation, the initial population of each strain was adjusted to approximately 10^5^ CFU mL^−1^. Fermentation was carried out at 30°C in Minifor 2BIO fermenters. To simulate a dairy environment, the initial pH of the modified MRS medium was set to 6.8. To simulate shubat fermentation, reconstituted camel milk was used as a model matrix. Every experiment was performed twice.

#### 2.3.4 Plate-counting methods for calibration curve

The homogenized samples were serially diluted ten times in a sterile 9 g⋅L^−1^ NaCl solution. For *L. helveticus* SH4 and *K. marxianus* Y45, 0.1 mL aliquots were plated on MRS agar and Sabouraud agar, respectively. For co-culture samples, LAB and yeast colonies were separated according to medium acidification using MRS agar supplemented with 0.05 g⋅L^−1^ bromocresol green. While yeast colonies did not cause a color shift, LAB colonies caused the surrounding media to become yellow. For 48 h, plates were incubated at 37°C. The calibration coefficient was calculated by plotting CFU counts against the corresponding OD600 values and turbidity readings obtained using the Dencytee Arc in-line optical turbidity/cell-density sensor. Continuous turbidity measurements were converted into viable-cell concentrations (CFU⋅mL^−1^) using this coefficient, and the resulting growth curves served as the basis for kinetic model fitting. The turbidity-viable count relationship was linear over the range examined, with *R*^2^ = 0.88 for both strains (*n* = 16 for SH4 and *n* = 9 for Y45).

### 2.4 Chemical analyses

The carbohydrate contents and the production of lactic acid and ethanol were measured by HPLC with separation on an Aminex HPX-87H column (Bio-Rad Laboratories, Inc., Hercules, CA, United States). Detection was performed using a refractive index detector for the ethanol, carbohydrates and lactic acid detection. Aliquots of 20 μL were injected and elution was performed at 30°C with 5 mM sulfuric acid at a flow rate of 0.6 mL⋅min-1. Samples were filtered through a 0.45 μm pore size filter before injection.

### 2.5 Mathematical modeling

#### 2.5.1 Logistic growth model

Equation 1 led to the selection of the logistic growth model (Delhalle et al., 2012) to explain microbial proliferation:


$$\left\{ \begin{array}{c} \frac{d\,N\,t}{d\,t}=0\,i\,f\,t\leq \lambda \\ \frac{d\,N\,t}{d\,t}=\mu \,m\,a\,x\,\text{Nt}\,\left(1-\frac{N\,t}{N\,m\,a\,x}\right)\,i\,f\,t>\lambda \end{array}\right.,$$ (1)

where μmax is the maximum growth rate (h-1), λ is the lag time (h), and Nt and Nmax (CFU/mL) are the microbial population levels at time t and at the end of the growth curve, respectively.

Equation (2) uses the gamma concept model (Zwietering et al., 1993) as a supplementary model to explain how temperature and pH affected the maximum growth rate (*μmax*).


$$\mu_{m\,a\,x}=\mu_{o\,p\,t}\times \gamma \,\left(T\right)\times \gamma \,\left(\mathrm{pH}\right)$$ (2)

with values of γ falling between 0 and 1.

The cardinal temperature and pH model (CTPM) introduced by (Rosso et al., 1995) was used to explain the influence of pH on μmax, as shown in Equation (3):


$$C\,M_{n}\,\left(X\right)=\frac{\begin{array}{c} X\leq X_{m\,i\,n};0\\ {\left(X-X_{m\,i\,n}\right)}^{n}\,\left(X-X_{m\,a\,x}\right) \end{array}}{\begin{array}{c} {\left(X_{o\,p\,t}-X_{m\,i\,n}\right)}^{n-1}\{\left(X_{o\,p\,t}-X_{m\,i\,n}\right)\left(X-X_{o\,p\,t}\right)-\\ \left(X-X_{m\,a\,x}\right)\left[\left(n-1\right)X_{o\,p\,t}+X_{m\,i\,n}-nX\right]\}\\ X\geq X_{m\,a\,x};;0 \end{array}}$$ (3)

where X represents environmental factors such pH and temperature, and n values are 1 for pH and 2 for temperature.

#### 2.5.2 Restricting the substrate

The modified Monod equation was used to model microbial growth kinetics (Monod, 1949; Kondybayev et al., 2023) (Equation 4):


$$\mu =\mu_{\text{max}}\,\frac{\text{S}}{\text{Ks}+\text{S}}+\mu_{\text{max}}\,0,$$ (4)

where μ is the specific growth rate, *μmax* is the maximum specific growth rate, μmax0 is the maximum specific growth rate in the absence of a limiting substrate, S is the concentration of the limiting substrate for growth, and Ks is the “half-velocity constant”—the value of S when μ/μmax = 0.5—was selected to relate growth rates to different types of limiting substrates.

#### 2.5.3 Modeling of metabolite production

The Luedeking and Piret equation was simplified by setting the kinetics of metabolite synthesis to be directly proportional to yeast growth, as indicated in Equation (5) (Munanga et al., 2016).


$$\frac{d\,\left(\mathrm{metabolite}\right)}{\text{dt}}=\frac{\text{Y}\,\left(\text{metabolite}\right)}{\text{N}}\times \mu \,\text{N}$$ (5)

where N is the population, μ is the growth rate, and Y(metabolite)/N is the yield for ethanol production over population.

#### 2.5.4 Fitting the model and estimating its parameters

All experiments were performed in duplicate (*n* = 2), and replicate measurements at each sampling point were averaged before model fitting. Model fitting and parameter estimation were performed by nonlinear least-squares minimization using the Solver add-in in Microsoft Excel. Experimental variability is presented as mean ± standard deviation (SD), whereas the uncertainty of the fitted model parameters is reported as standard error (SE) and was calculated using SolverAid. Standard errors of regression lines were obtained using the Data Analysis add-in in Microsoft Excel. Values reported as mean ± SE are indicated by an asterisk. Goodness of fit was assessed by the coefficient of determination (*R*^2^) and the root mean square error (RMSE) calculated between observed and predicted values; for the primary growth models these statistics were computed on ln-transformed cell concentrations, and for the secondary and Monod models on the maximum specific growth rates.

## 3 Results and discussion

### 3.1 Growth characteristic of *L. helveticus* and *K. marxianus*

#### 3.1.1 Modeling microorganism growth

**FIGURE 1:**
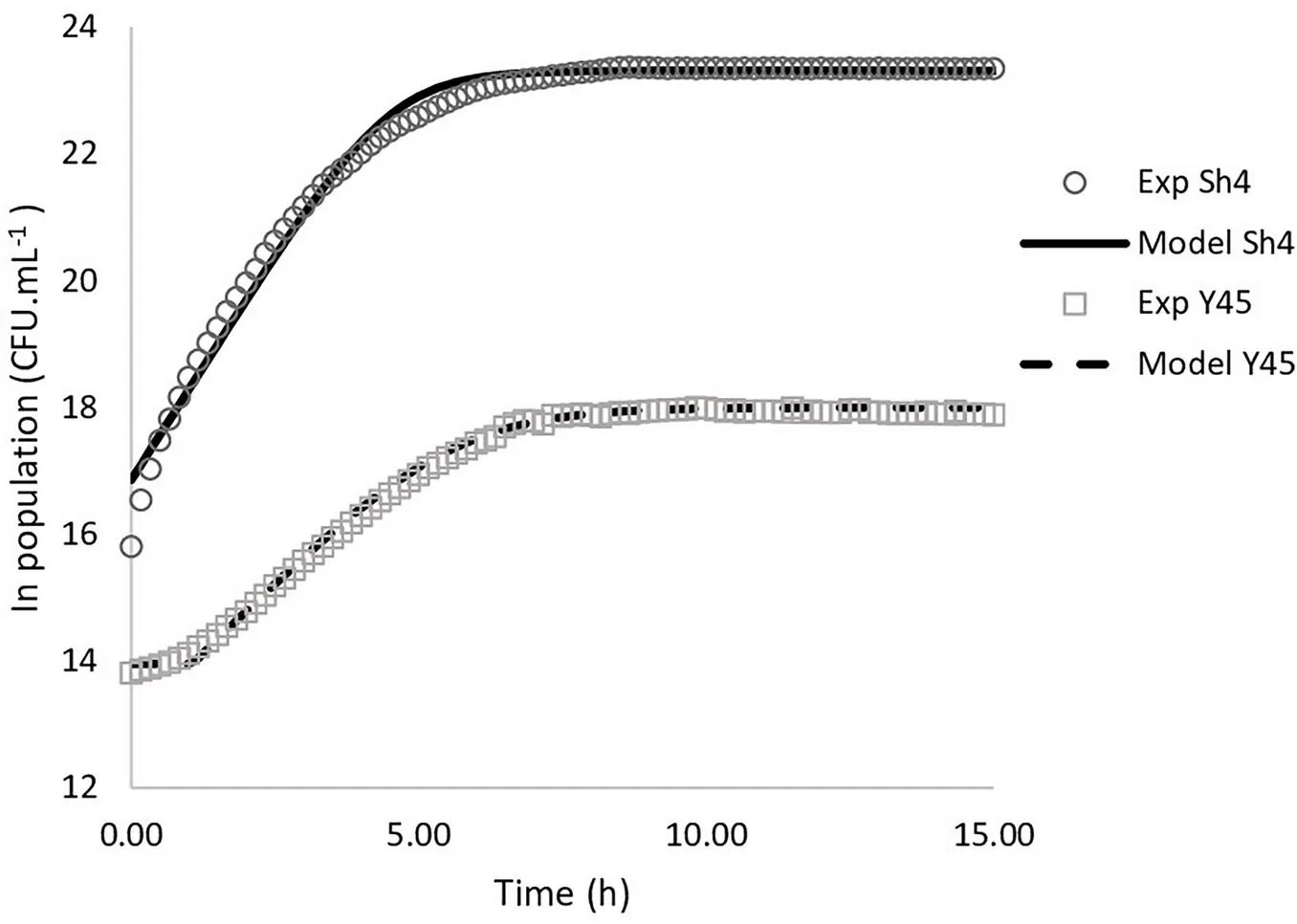
Primary models of L *helveticus* (SH4) and K *marxianus* (Y45).

The basic growth curves demonstrated that both unique shubat isolates could develop fast under controlled culture conditions. The standard logistic model did not describe both isolates equally well; for *L. helveticus* SH4, a systematic deviation was evident during the transition to the stationary phase (Figure 1). A more flexible Richards-type model (Richards, 1959) followed the data more closely, but returned a maximum specific growth rate of 3.0 h^–1^, corresponding to an implausibly short doubling time of roughly 14 min, inconsistent with the value measured independently in microplates by the serial-dilution method (Augustin et al., 1999). The reason is that μmax does not denote the same quantity in every growth model: in differential formulations like the Richards model, it is defined as the theoretical growth rate at infinite dilution (N→0) rather than the steepest slope of the observed exponential curve (Baranyi et al., 1993; Perni et al., 2005). Perni et al. (2005) demonstrated that models fitting identical growth data well can yield raw μmax values differing by 20–60% for this reason alone. A closer statistical fit therefore does not guarantee a more realistic growth rate. The logistic model was therefore retained because it uses fewer parameters, yields growth rates that align with empirical measurements and provide robust inputs for secondary modeling. Fitted with the logistic model, the primary growth curves described the experimental data closely, with *R*^2^ = 0.990 and RMSE = 0.157 ln CFU⋅mL^−1^ for *L. helveticus* SH4 at 35°C, and *R*^2^ = 0.977 and RMSE = 0.173 ln CFU⋅mL^−1^ for *K. marxianus* Y45 at 38 °C. The *μmax* value for *L. helveticus* SH4 was higher than that reported by Bolívar-Jacobo et al. (2023), who reported a maximum growth rate of 0.395 ± 0.007 h^−1^ for the untreated *L. helveticus* culture and 0.410 h^−1^ following ultrasonic treatment with 20–30% amplitude. Schepers et al. (2002) found an optimal μmax of 0.7 h^−1^ for *L. helveticus* R211 during pH-controlled batch culturing in whey permeate/yeast extract medium at pH 5.5. The increased μmax found for *L. helveticus* SH4 might be attributed to strain-specific physiological features, its origin from traditional shubat, changes in substrate composition and nutritional availability, and the utilization of controlled bioreactor conditions in the current investigation. Similar behavior was seen with *K. marxianus* Y45. Fonseca et al. (2007) reported a μmax of 0.56 ± 0.02 h^−1^ for *K. marxianus* ATCC 26548 during aerobic batch culturing on glucose, whereas Urit et al. (2013) demonstrated that *K. marxianus* DSM 5422 exhibited a maximum growth rate of 0.75 h^−1^ at 40°C. In comparison, *K. marxianus* Y45 *μmax* of 0.90 h^−1^ indicates a significant growth capability under studied conditions.

#### 3.1.2 Modeling the effect of pH and temperature on strains

To assess the impact of environmental conditions on shubat isolate development, the maximum specific growth rates obtained at various pH and temperature values were fitted using the cardinal pH and temperature model. The model characterized the bell-shaped responses of both strains, indicating that the growth rate increased to an optimum and subsequently decreased when the pH or temperature moved outside the favorable range. Agreement between observed and predicted maximum specific growth rates was good in all four cases: for SH4, *R*^2^ = 0.96 and RMSE = 0.08 h^−1^ for the pH model and *R*^2^ = 0.99 and RMSE = 0.05 h^−1^ for the temperature model; for Y45, *R*^2^ = 0.97 and RMSE = 0.04 h^−1^ for the pH model and *R*^2^ = 0.92 and RMSE = 0.07 h^−1^ for the temperature model.

**FIGURE 2:**
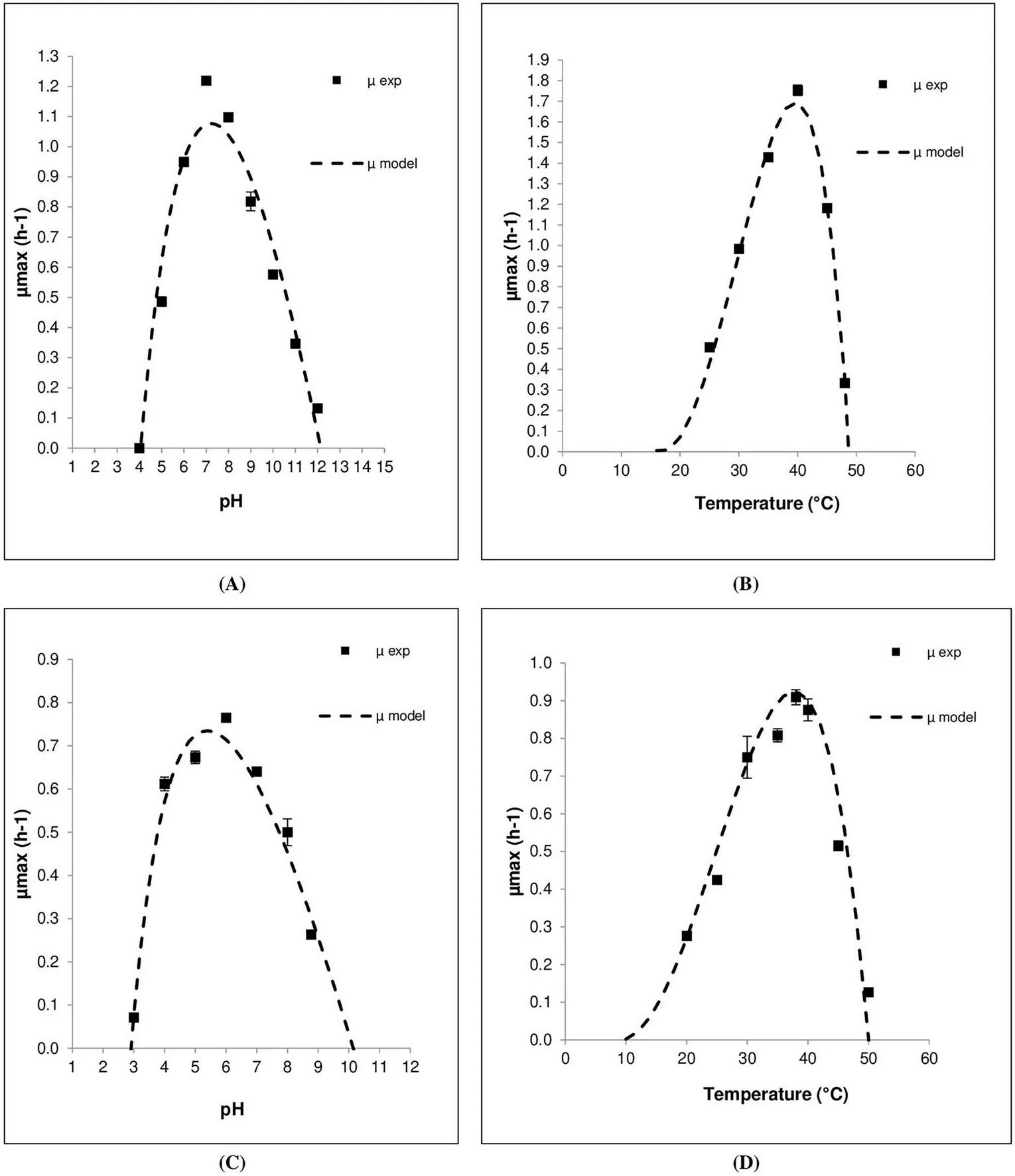
Secondary models of shubat microorganisms: **(A)** SH4 pH model, **(B)** temperature model, **(C)** Y45 pH model, **(D)** Y45 temperature model.

*L. helveticus* SH4’s optimum pH and temperature were 7.22 ± 0.11 (Figure 2A) and 39.66 ± 0.38°C (Figure 2B), respectively. The strain’s pH ranged from 4.05 ± 0.05 to 12.15 ± 0.17, with estimated temperature limits of 16.86 ± 1.33 and 48.76 ± 0.22°C. These findings support *L. helveticus* SH4’s thermotolerance and demonstrate that this strain develops most actively in near-neutral pH conditions. In studies by Zang et al. (2021), the optimal temperature and pH for *Lactobacillus helveticus* was shown to be 42°Ñ and a pH of 6.5, which showed high growth of the microorganism. The temperature optimum of Y45 is consistent with previous studies on *K. marxianus* thermotolerance. Urit et al. (2013) found that *K. marxianus* DSM 5422 could grow between 7 and 47°C, with a maximum growth rate at 40°C. In the current investigation, the estimated Topt was 37.78°C (Figure 2D), which was significantly lower but still within the thermotolerant range described for this species. The pH optimum of Y45 was higher than that reported by Lo et al. (2021), who found optimal growth of *K. marxianus* at pH 4.18 when lactate was the sole carbon source. This variation may be explained by the carbon supply and culture medium, as lactate-based media impose different physiological circumstances than carbohydrate-rich media. As a result, *K. marxianus* Y45’s pH optimum of 5.39 (Figure 2C) may be more appropriate for its involvement in fermented milk, where yeasts interact with sugars, organic acids, and LAB-derived metabolites.

#### 3.1.3 Effect of carbohydrate source on strain growth

*L. helveticus* SH4 showed similar maximum specific growth rates for all three carbo-hydrates examined. The μopt values for glucose, galactose, and lactose were 0.87 ± 0.01 h^−1^, 0.87 ± 0.01 h^−1^, and 0.90 ± 0.02, respectively. These findings show that *L. helveticus* SH4 can grow efficiently on both monosaccharides and lactose, which is compatible with the dairy-associated physiology of *L. helveticus* (Chelladhurai et al., 2023). The Ks values varied substantially between substrates. *L. helveticus* SH4 has Ks values of 0.04 ± 0.10 g L^−1^ for glucose, 0.86 ± 0.25 g L^−1^ for galactose, and 0.10 ± 0.22 g L^−1^ for lactose. Since Ks reflects the substrate concentration at which the growth rate reaches 50% of its maximum value, the higher Ks value for galactose implies that *L. helveticus* SH4 needed a greater galactose concentration to attain its maximum growth rate. Although SH4 can grow on galactose with a μopt similar to glucose and lactose, it appears to be less beneficial in terms of substrate affinity. This might be explained by the fact that galactose takes more transport and metabolic conversion steps than glucose (Iskandar et al., 2019). Recent genomic study revealed that milk-associated *L. helveticus* strains had entire gene sets implicated in the Leloir pathway for galactose processing (Kido et al., 2021). In *K. marxianus* Y45, glucose had the greatest growth rate (μopt = 0.46 ± 0.01 h^−1^). Growth on galactose and lactose was lower but comparable, with μopt values of 0.39 ± 0.02 h^−1^ and 0.39 ± 0.01 h^−1^, respectively. This suggests that glucose was the most effective carbon source for fast *K. marxianus* Y45 development, whereas galactose and lactose also promoted growth, but at a less rapid pace. A similar trend was documented for *K. marxianus* strains fed on glucose, galactose, and lactose (De Micco et al., 2025). *K. marxianus* Y45 showed Ks values of 0.07 ± 0.09 g/L^−1^ for glucose, 1.26 ± 1.35 g/L^−1^ for galactose, and 0.23 ± 0.29 g/L^−1^ for lactose. As with SH4, galactose had the greatest Ks value. This implies that galactose may be a less desirable substrate for *K. marxianus* Y45 in terms of perceived affinity. The reliability of the estimated Ks values requires explicit qualification. For both strains the standard errors of Ks exceeded, or were of the same order as, the estimates themselves for glucose and lactose, and this is a direct consequence of the experimental design rather than of a poor model fit. Because the fitted Ks values (0.04–0.23 g⋅L^−1^ for glucose and lactose) lie well below the lowest substrate concentration tested (0.5 g⋅L^−1^), the Monod response was effectively saturated across the entire experimental range, leaving little curvature from which Ks can be identified. The consequence is visible in the fit statistics: RMSE remained low throughout (0.011–0.042 h^−1^, equivalent to 2–5% of μopt), confirming that the fitted curves reproduce the data closely, whereas *R*^2^ was high only for galactose in SH4 (*R*^2^ = 0.91), the single substrate whose Ks (0.86 g⋅L^−1^) is large enough to produce measurable curvature within the concentration range examined. For the remaining substrate–strain combinations *R*^2^ ranged from 0.04 to 0.36, reflecting the near-absence of variance in μ across the tested concentrations rather than a failure of the Monod description. The Ks values reported here should therefore be interpreted as upper-bound estimates indicating high apparent substrate affinity, and not as precisely resolved constants. Beniwal et al. (2017) found that *K. marxianus* may thrive on galactose-containing medium and hydrolyzed cheese whey, with μmax values similar to those observed for *K. marxianus* Y45 in the current investigation.

### 3.2 Lactic acid and ethanol production

**FIGURE 3:**
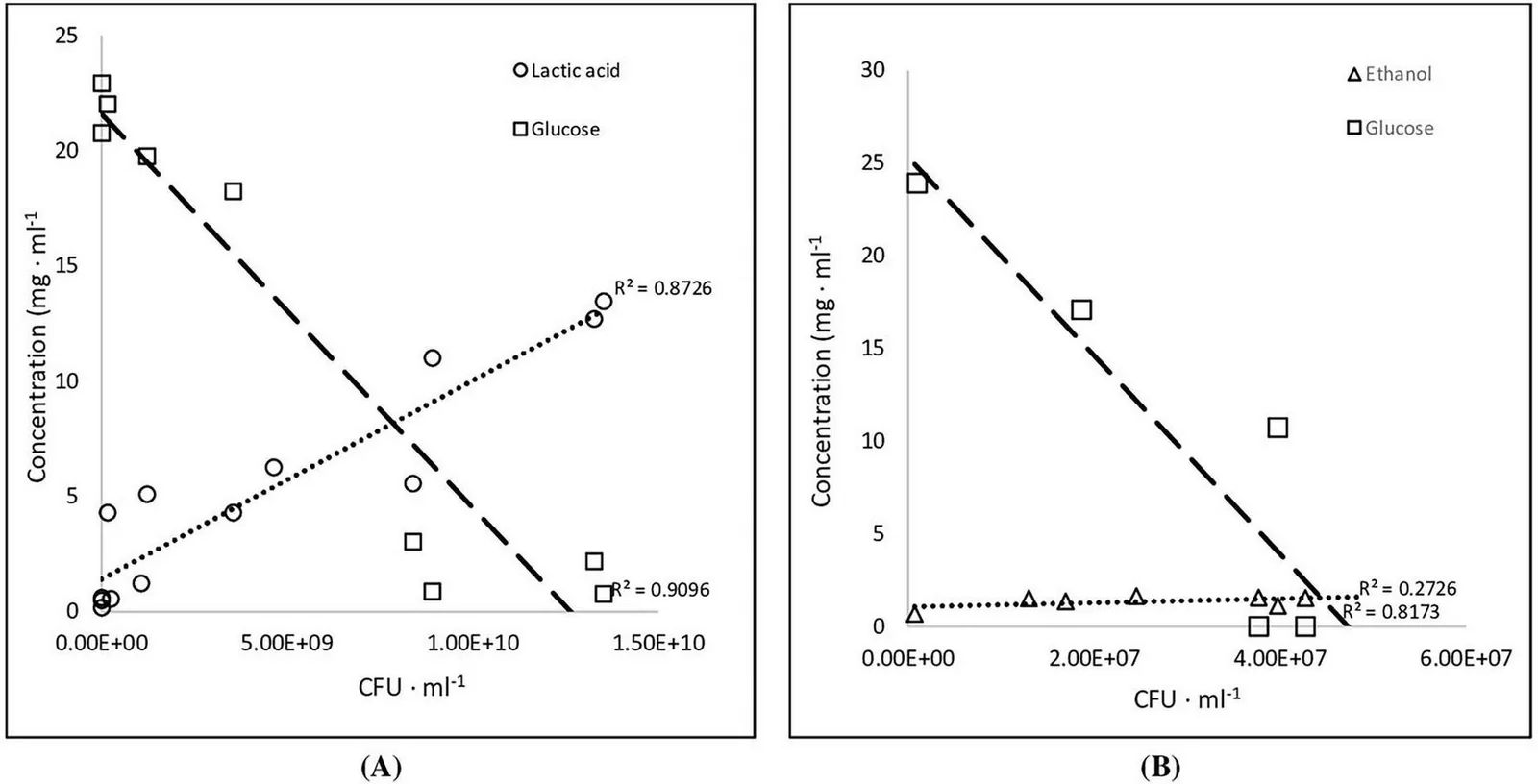
Metabolite production of shubat microorganisms. **(A)** SH4 lactic acid production. **(B)** Y45 ethanol production.

Lactic acid production was evaluated during the growth of *L. helveticus* SH4 in the fermenter (Figure 3A). During cultivation, glucose concentration decreased, while lactic acid concentration increased, indicating that *L. helveticus* SH4 mainly converted the available carbohydrate into lactic acid. The lactic acid production rate calculated for SH4 was 8.63 ± 0.9 × 10^−9^ mg⋅CFU^−1^. Since *L. helveticus* is described as a homofermentative, thermophilic starter bacterium commonly used in dairy fermentation (Chelladhurai et al., 2023), the predominance of lactic acid formation in the present experiment is consistent with the expected metabolic profile of this species. Thus, SH4 demonstrated homofermentative behavior under the tested cultivation conditions. Kylä-Nikkilä et al. (2000) cultured *L. helveticus* strains in a pH-controlled bioreactor using lactose-rich whey permeate medium, yielding 91.6 and 76.2% of lactic acid, respectively. In that investigation, lactic acid continued to accumulate into the late stationary phase, even after the substrate had been completely depleted. In the current study, by contrast, monitoring was terminated once turbidity measurements no longer indicated active bacterial growth, rather than being extended through the stationary phase. Because lactic acid can continue to accumulate after cell growth ceases and the substrate is exhausted, ending measurement at the turbidity plateau likely truncated the fermentation profile. Consequently, the lower apparent conversion reported for SH4 may reflect this premature sampling cutoff rather than a genuine limitation in the strain’s lactic acid-producing capacity. Ethanol production by *K. marxianus* Y45 did not exceed 1.5 g⋅L^–1^ (Figure 3B), indicating limited ethanol accumulation under the tested conditions. This relatively low production may reflect strain-specific metabolic characteristics and the influence of cultivation conditions on carbon distribution between biomass formation and fermentative metabolism.

### 3.3 Co-culture growth of *Lactobacillus helveticus* SH4 and *Kluyveromyces marxianus* Y45

**FIGURE 4:**
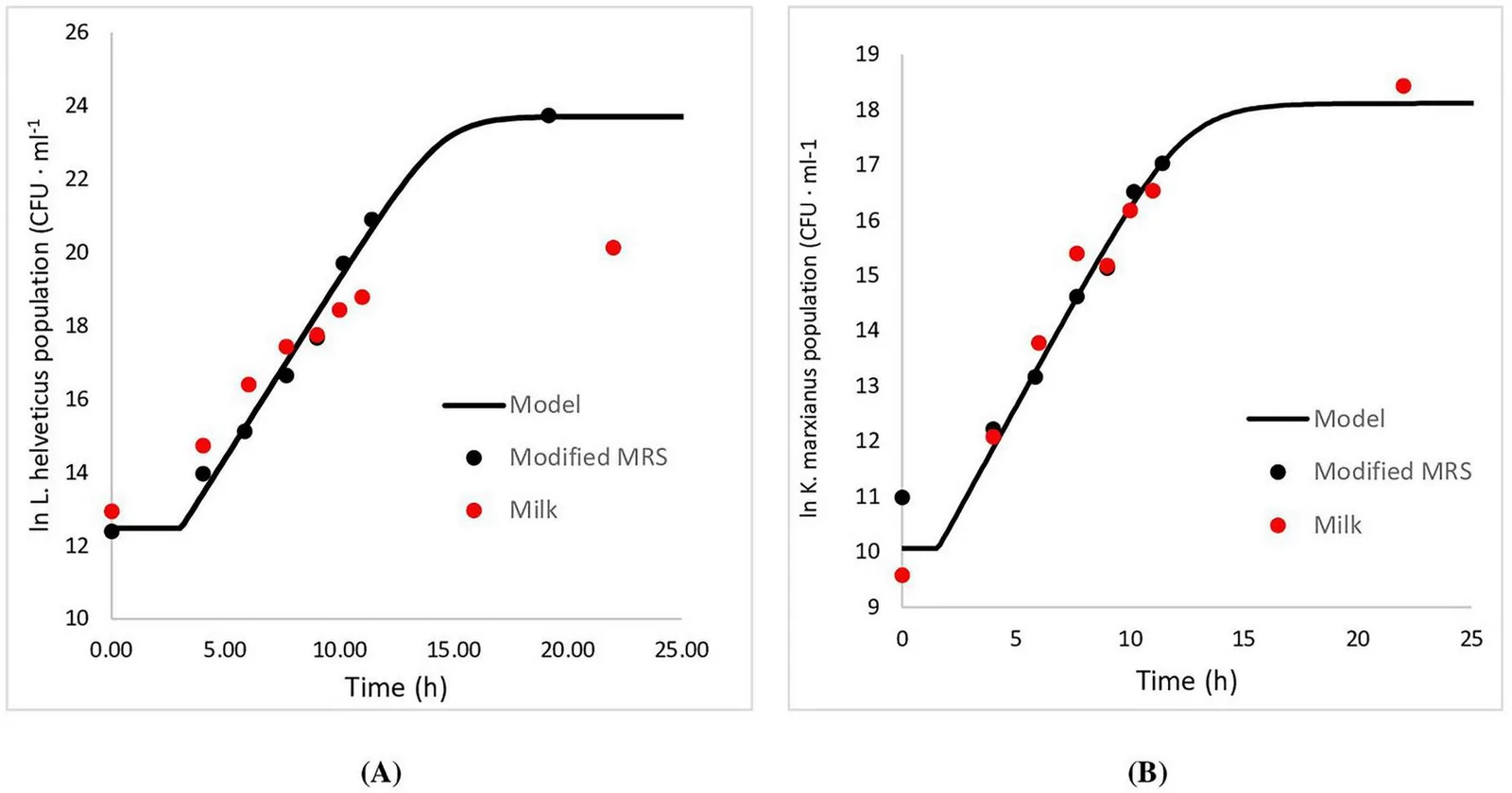
Coculture growth of microorganisms. **(A)** SH4 in modified MRS (black) and milk (red). **(B)** Y45 in modified MRS (black) and milk (red).

*The coculture of L. helveticus* SH4 and *K. marxianus* Y45 in modified MRS medium and reconstituted camel milk at 30°C is presented in Figure 4. Experimental growth profiles were compared with monoculture-based growth models to evaluate whether the interaction between the two strains affected their growth behavior during co-cultivation.

In modified MRS medium, the SH4 population followed the predicted model and reached approximately 2.0 × 10^10^ CFU⋅mL^−1^. In contrast, in reconstituted camel milk, the final SH4 population was lower, reaching approximately 5.5 × 10^8^ CFU⋅mL^−1^ (Figure 4A). For Y45, the final population reached around 108 CFU⋅mL^−1^ in both media and was generally consistent with the model predictions (Figure 4B).

The agreement between the monoculture-based model and the experimental data in modified MRS medium suggests that there was no clear antagonistic interaction between SH4 and Y45 under these conditions. However, the reduced growth of SH4 in reconstituted camel milk indicates that the milk matrix may have imposed additional nutritional or physicochemical limitations on bacterial growth. Previous studies on co-cultures of *L. helveticus* and *K. marxianus* have reported a reduction in *L. helveticus* growth compared with monoculture conditions, which was attributed to ethanol production by *K. marxianus* (Ahtesh et al., 2018). In contrast, Liu et al. (2026) reported a different interaction pattern, in which the lactobacilli exhibited growth comparable to that observed in monoculture, whereas *Kluyveromyces marxianus* showed reduced growth under co-culture conditions. Such discrepancies between studies may be attributable to strain-specific physiological characteristics and differences in metabolic interactions between the microbial partners. Together with the present results, these findings indicate that LAB–yeast interactions are strongly dependent on the strains used and on the fermentation matrix.

The early area of the curves indicates changes in the apparent adaption phase. However, the calculation of Tlag should be regarded with caution because lag time is dependent on the quantity and distribution of early sample sites. This problem has also been identified in fermentation modeling research (Munanga et al., 2016). As a result, the observed early phase variations are better analyzed as changes in the perceived adaptation phase rather than actual quantitative differences in Tlag. The co-culture results show that SH4 and Y45 were compatible in modified MRS, but the camel milk matrix had a significant impact on the final *L. helveticus* SH4 population.

### 3.4 Estimated model parameters

The estimated parameters for the growth models of *Lactobacillus helveticus* SH4 and *Kluyveromyces marxianus* Y45 are presented in Table 1.

**TABLE 1:** Resulting parameters of models.

Parameters	*Lactobacillus helveticus* (value ± SD/SE)	*Kluyveromyces marxianus* (value ± SD/SE)
pH min	4.05 ± 0.05	2.90 ± 0.08
pH max	12.15 ± 0.17	10.15 ± 0.46
pH opt	7.22 ± 0.11	5.39 ± 0.23
T min (°C)	16.86 ± 1.33	9.13 ± 3.08
T max (°C)	48.76 ± 0.22	50.03 ± 0.33
T opt (°C)	39.66 ± 0.38	37.78 ± 0.71
μ_*opt*_ (h^–1^)	1.91 ± 0.07	0.92 ± 0.04
Glucose μ_*opt*_ (h^–1^)	0.87 ± 0.01	0.46 ± 0.01
Glucose Ks (g L^–1^)	0.04 ± 0.10	0.07 ± 0.09
Galactose μ_*opt*_ (h^–1^)	0.87 ± 0.01	0.39 ± 0.02
Galactose Ks (g L^–1^)	0.86 ± 0.25	1.26 ± 1.35
Lactose μ_*opt*_ (h^–1^)	0.90 ± 0.02	0.39 ± 0.01
Lactose Ks (g L^–1^)	0.10 ± 0.22	0.23 ± 0.29
Lactic acid production * (10^–9^ mg⋅CFU^–1^)	8.63 ± 0.9	0
Ethanol production* (10^–8^ mg⋅CFU^–1^)	0	1.11 ± 0.80

*Values are expressed as mean ± standard error (SE).

Although SH4 and Y45 were isolated from shubat, the parameters in Table 1 are strain-intrinsic descriptors rather than matrix-specific ones. The cardinal values (Tmin, Topt, Tmax, pHmin, pHopt) and the Monod constants for glucose, galactose, and lactose were estimated in defined media and characterize the organisms themselves; a change of fermentation matrix acts primarily as a multiplicative correction on μopt rather than a redefinition of these constants. Whether these constants transfer to bovine or ovine systems remains to be established, since matrix effects on μopt were not quantified in the present work; the reduced final SH4 population observed in reconstituted camel milk indicates that matrix influence cannot be assumed to reduce to a single multiplicative correction.

Several limitations should be considered when interpreting these results. All experiments were performed in duplicate (*n* = 2), which is sufficient for parameter estimation but limits the statistical characterisation of experimental variability. As discussed above, the half-saturation constants for glucose and lactose are poorly resolved because the substrate concentrations tested lay above the saturation threshold of the Monod response. Finally, and most importantly for the transfer of these results to practice, cultivations were carried out in stirred bioreactors at 240 rpm with automatic pH control, whereas traditional shubat is fermented without continuous agitation and without pH regulation (Bilal et al., 2024). Agitation at this level maintains a homogeneous, comparatively well-aerated environment and prevents the progressive acidification that characterizes spontaneous fermentation, both of which favor higher growth rates and may alter the balance between lactic acid and ethanol formation. The kinetic parameters reported here should therefore be regarded as strain descriptors obtained under controlled conditions, representing upper bounds for growth performance rather than values directly transferable to artisanal shubat production.

## 4 Conclusion

This study provides strain-specific kinetic parameters describing the growth and metabolic activity of *Lactobacillus helveticus* SH4 and *Kluyveromyces marxianus* Y45 isolated from traditional shubat. The secondary models showed that the two strains had distinct optimal growth conditions. *L. helveticus* SH4 grew optimally under near-neutral pH and thermophilic conditions, whereas *K. marxianus* Y45 preferred a more acidic pH and a slightly lower optimal temperature. These differences indicate that the two microorganisms may contribute differently to the fermentation process depending on pH, temperature, and medium composition.

The substrate-dependent growth results showed that SH4 was able to grow efficiently on glucose, galactose, and lactose, supporting its adaptation to milk-associated carbohydrates. Y45 showed a higher growth rate on glucose, but also maintained growth on lactose and galactose, confirming its capacity to participate in milk-sugar utilization. Metabolite analysis further demonstrated a functional separation between the two strains: SH4 mainly contributed to lactic acid formation, while Y45 produced ethanol. This metabolic division is important for shubat fermentation, where the balance between acidification and ethanol formation contributes to the characteristic acidic-alcoholic profile of the beverage.

Co-culture experiments showed that *Lactobacillus helveticus* SH4 and *Kluyveromyces marxianus* Y45 were compatible in modified MRS medium, where both strains followed their monoculture-based growth trends and no clear antagonistic interaction was observed. In reconstituted camel milk; however, SH4 reached a lower final population, whereas Y45 maintained active growth. This indicates that the fermentation matrix had a stronger effect on bacterial population development than on yeast growth under the tested conditions. The results demonstrate that microbial interaction in shubat depends not only on strain compatibility but also on the physicochemical and nutritional properties of camel milk.

The principal contribution of this study is the quantitative kinetic characterization of two autochthonous shubat isolates in a camel-milk system. Although the growth of L. helveticus and K. marxianus has been investigated previously, strain-specific parameters for SH4 and Y45 under different pH, temperature, and carbohydrate conditions, together with their metabolite-production characteristics, have not previously been reported.

The obtained kinetic parameters provide a quantitative basis for further optimization and standardization of shubat fermentation. These data may be useful for designing controlled starter cultures and improving the reproducibility of traditional fermented camel milk products. Further validation under pilot-scale and industrial fermentation conditions is required to confirm the applicability of the proposed models in practical production systems.

## Data Availability

The datasets presented in this study can be found in online repositories. The names of the repository/repositories and accession number(s) can be found at: https://www.ncbi.nlm.nih.gov/genbank/, PZ466835; https://www.ncbi.nlm.nih.gov/genbank/, PZ466841.
